# Willingness to pay for improved solid waste management and associated factors among households in Injibara town, Northwest Ethiopia

**DOI:** 10.1186/s13104-019-4433-7

**Published:** 2019-07-12

**Authors:** Selamawit Mulat, Walelegn Worku, Amare Minyihun

**Affiliations:** 1 Planning and Monitoring and Evaluation Officer of Awi Zonal Health Department, Amhara Regional Health Bureau, Injibara, Ethiopia; 2 Department of Environmental and Occupational Health and Safety, Institute of Public Health, College of Medicine and Health Sciences, University of Gondar, Gondar, Ethiopia; 3 Department of Health Systems and Policy, Institute of Public Health, College of Medicine and Health Sciences, University of Gondar, P.o. Box:196, Gondar, Ethiopia

**Keywords:** Willingness to pay, Solid waste, Tobit model, Injibara

## Abstract

**Objective:**

Globally, cities generate 1.3 billion tons of solid waste per year, amounting to a rate of 1.2 kg per person per day. Solid waste management is an important challenge to both the developed and developing countries. This study aimed to assess the willingness to pay for the improved solid waste management and associated factors among households in Injibara town, Ethiopia.

**Results:**

A total of 903 household heads participated in the study. The finding indicated that 81.06% were willing to pay for the service. The average amount of money the participants would be willing to pay per month was 29.7 ETB ($1.07)). The study revealed that sex (β = 3.24, (95% CI 1.98, 4.50)), age (β = − 0.09: 95% CI − 0.19, − 0.01), educational status (β = 6.19: 95% CI 3.54, 8.84), occupation (β = 2.43: 95% CI 1.009, 3.86), amount of solid waste generated (β = 1.74: 95% CI 0.19, 3.29), distance from dump site (β = 1.58: 95% CI 0.45, 2.72), satisfaction with the existing service (β = 3.89, (95% CI 2.75, 5.06) and wealth status (β = 2.43: 95% CI 1.0, 3.86) were statistically significant. Therefore, the level of premium load should consider the amount of waste generated, wealth status and the distance from the dump site.

**Electronic supplementary material:**

The online version of this article (10.1186/s13104-019-4433-7) contains supplementary material, which is available to authorized users.

## Introduction

Globally, the amount of municipal solid waste is growing faster than the rate of urbanization. In 2012, the world’s cities generated 1.3 billion tons of solid waste per year, amounting to a rate of 1.2 kg per person per day. With rapid population growth and urbanization, municipal waste generation is expected to rise to 2.2 billion tons by 2025. In sub-Saharan Africa, waste generation is approximately 62 million tons per year ranging from 0.09 to 3.0 kg per person per day, with an average of 0.65 kg/capita/day [1–4].

In developing countries, waste management requires a large expenditure of 30–50 percent of municipal operational budgets. However, cities collect only half of the wastes generated [5, 6].

In Ethiopia, the per capita amount of waste generated ranges from 0.28 to 0.83 kg/person/day [7] and it lacks the financial resources and institutional capacity to provide the needed municipal infrastructure for adequate solid waste management (SWM) [8]. In Ethiopia, the government realized that it is impossible to address the problem of environment, particularly solid waste management, without involvement of local communities [9].

Traditional mode of transportation, irregular waste picking up program, having few required equipment and no fence for dumpsites are the prevailing problems in Ethiopia [10]. To minimize these problem the participation of local communities or service receivers is important in providing solutions to problems of SWM [8].

Households which are the primary producers of solid waste and suffer from the effects of uncollected solid waste should be able to participate in improving SWM. Accordingly, the contribution of urban dwellers on SWM service plays a great role for better improvement of SWM at the community [11]. However, there is limited evidence on the willingness to pay (WTP) for improved solid waste management (ISWM) and associated factors in the study area. Therefore, this study aimed to assess the WTP for ISWM and associated factors among households in Injibara town, Ethiopia.

## Main texts

### Methods

Community based cross-sectional study was conducted from March to April, 2018 in Injibara town, 447 km away from Addis Ababa. It is the administrative center of the Agew Awi Zone in the Amhara Region. According to the town Administration report, the town has a total population of 35,846 and 7169 households. It is divided into five administrative kebeles (the smallest administrative units in the country). There are two private waste collectors and three waste dumping sites in the town.

The source population of this study was all household heads living in Injibara town, and the study population of this study was all household heads in the randomly selected kebeles in Injibara town. Household heads residing in the selected kebeles were included in the study; and those household heads lived for less than 6 months in the selected kebeles were excluded for the study.

The sample size was calculated by using single population proportion formula assuming: 95% confidence level, 3% margin of error, and 83.5% proportion of WTP(P) [12], 10% non-response rate, and 1.5 design effect. Accordingly, the final sample size was 970 household heads.

Multi-stage sampling procedure was used to select the study participants. In the first stage, three out of five kebeles of the town were randomly selected. In the second stage, the total sample size was proportionally allocated to the number of households in the selected kebeles. Finally, systematic random sampling technique was used to select every 4th participant.

#### Study variables

The dependent variable of the study was WTP for ISWM. The independent variables were sociodemographic and socioeconomic characteristics (age, gender, marital status, educational status, occupation, family size, time spent in the area, household ownership, wealth status), awareness about SWM, service-related factors (quantity of waste generated, satisfaction with current service, distance of waste disposal site). WTP for ISWM service is measured by bid contingent valuation method (CVM) where the respondent was first asked whether they would be willing to pay initial bid of specific amount (20 Birr) and probing the question using a higher or lower bid value depending on the respondent’s response to the first question until the maximum amount of money participants were willing to pay.

A pretested and standardized interviewer administered and semi-structure questionnaire was used to collect the data. The pretest was done on 48 individuals in Kebele 02, one of the kebeles found in the town.

After appropriate coding, the data were entered using Epi data version 7 software, and exported to STATA version 14 for further analysis.

Tobit model was used to analyse factors associated with WTP and the maximum amount of money that individuals were willing to pay. This model reveals both the probability of WTP and the maximum amount of money the respondents are willing to pay.

$${\text{y}} = \left\{ {\begin{array}{*{20}l} {1\quad {\text{i}}\;{\text{MWTP}} = {\upbeta \text{o}} + {\upbeta^{\prime}}{\text{Xi}} + {\text{e}} > 0} \\ {0\quad {\text{if}}\;{\text{MWTP}} \le 0} \\ \end{array} } \right.$$

where Y: outcome, X: predictor, βo: slope, β′: coefficient, E: error term, 0: no, 1: yes, and MWTP: maximum willingness to pay.

The model estimates marginal effect of an explanatory variable on the expected value of the dependent variable. To be free from serious data error, the assumptions of Tobit model such as normality, linearity, multicollinearity and equal variance were tested. A p-value  ≤ 0.05 was used to determine statistical significance.

### Results

#### Socio-demographic and economic characteristics of study participants

A total of 903 (with a response rate of 93%) household heads participated in the study. The study revealed that, among the study participants, 71% were males, 48.17% were Amhara, 89% were orthodox and 83% were married. The mean age, mean family size, and mean time of stay in the study town of the participants were 38 ± 7.89 years, 4 ± 1.25, and 14 ± 9.2 years, respectively (Table 1).

**Table 1:** Socio-demographic and economic characteristics of the study participants in Injibara town Northwest Ethiopia, 2018 (n = 903)

Variable	Description	Frequency (%)
Sex	Male	640 (70.87)
	Female	263 (29.13)
Age (years)	< 30	138 (15.3)
	30–39	484 (53.6)
	40–49	209 (23.1)
	≥ 50	72 (8)
Religion	Orthodox	802 (88.82)
	Muslim	36 (3.99)
	Protestant	65 (7.20)
Ethnicity	Amhara	435 (48.17)
	Awi	434 (48.06)
	Tigre	17 (1.88)
	Oromo	17 (1.88)
Marital status	Married	751 (83.17)
	Single	108 (11.96)
	Widowed	17 (1.88)
	Divorced	27 (2.99)
Occupation	Civil servants	422 (46.73)
	Merchants	240 (26.58)
	House wife	106 (11.74)
	Daily labour	39 (4.32)
	Self-employer	96 (10.63)
Education	Can’t read and write	31 (3.43)
	Can read and write	79 (8.75)
	Primary school (1–8)	157 (17.39)
	Secondary school (9–12)	214 (23.70)
	College and above	422 (46.73)
House ownership	Yes	628 (69.55)
	No	275 (30.45)
Family size	1–4	637 (70.5)
	≥ 5	266 (29.5)
Time of stay (years)	< 8	247 (27.4)
	8–13	225 (24.9)
	14–20	257 (28.5)
	> 20	174 (19.3)
Wealth of the household	Poor	301 (33.33)
	Medium	302 (33.44)
	Rich	300 (33.22)

#### Service and solid waste related characteristics of households

Six hundred forty-four (71%) of household heads used the existing SWM service, 14.6% of respondents disposed wastes at community dump site, 10% burnt in their compounds and 4% threw outside their compound into rivers and sewerage lines. Fifty nine percent of the study participants were satisfied with the existing service. Besides, 55% of participants lived near the waste dump site and 63% of participants generated one sack of solid waste per week. Among household heads 89% had awareness about the environment (Table 2).

**Table 2:** Service and solid waste related characteristics of households in Injibara town Northwest Ethiopia, 2018 (n = 903)

Variable	Description	Frequency (%)
Distance of the dump site	Near (< 1 km)	493 (54.60)
	Far (≥ 1 km)	410 (45.40)
Amount of solid waste generated per week	< 1 sack of 50 kg	239 (26.47)
	1 sack of 50 kg	571 (63.23)
	2 sack of 50 kg	93 (10.30)
Perceived satisfaction about the service (n = 644)	Not very satisfied	44 (6.83)
	Not satisfied	205 (31.83)
	Neutral	14 (2.17)
	Satisfied	343 (53.26)
	Very satisfied	38 (5.90)
Household awareness about the environment	Good awareness	804 (89.03)
	Poor awareness	99 (10.97)

#### Willingness to pay (WTP) for improved solid waste management service

In this study, 81.06% (95% CI 78.5, 83.6) of participants were willing to pay for improved SWM. The mean(± SD) amount of money the study participants were willing to pay per month was 29.7 (95% CI = 29.08, 30.37) ETB (± 8.89) or 1.07 $USD (Additional file 1: Figure S1).

#### Factors associated with willingness to pay for improved solid waste management

This study showed that male participants were WTP 3.24 ETB more than those female participants by holding other variables constant (β = 3.24, 95% CI (1.98, 4.50), dx/dy = 0.000084).

As the age of participant increased by 1 year their WTP for SWM service decreases by − 0.09 ETB holding other variables constant (β = − 0.09, 95% CI (− 0.194, − 0.006), dx/dy = − 2.43e−06). Additionally, those who were satisfied with the service were WTP 3.9 ETB more than those who were dissatisfied with holding other variables constant (β = 3.89, 95% CI (2.75, 5.06), dx/dy = 0.0001).

Participants with secondary and college education were WTP 6.2 ETB and 3.5 ETB more than those who were not educated with holding other variables constant (β = 6.19, 95% CI (3.54, 8.84), dx/dy = 0.00006).

Study participants who were living far from the dump site were WTP 1.6 ETB more than those who were near the dump site with keeping other variables constant (β = 1.58, 95% CI (0.45, 2.72), dx/dy = 0.00004).

Study participants who generate one or more sack of waste per week were WTP 1.7 ETB more than those who generate less than one sack with holding other variables constant (β = 1.74, 95% CI (0.19, 3.29), dx/dy = 0.00004).

Households with better income were WTP 2.43 ETB more than those who were poor with holding other variables constant (β = 2.43, 95% CI (1.009, 3.86), dx/dy = 0.00003).

Furthermore, participants who were employed in civil sectors were willing to pay 3.4 ETB than those who were unemployed with holding others constant (β = 3.43, 95% CI (1.28, 5.58), dx/dy = 0.00005) (Table 3).

**Table 3:** Tobit regression results of factors affecting willingness to pay for improved waste management services in Injibara town North west Ethiopia, 2018 (n = 903)

Parameter for MWTP	Coefficients	Standard error	t-value	p-value	95% CI	Marginal effect (dy/dx)
Sex (ref. female)						
Male	3.24	0.64	5.06	0.000***	1.98, 4.50	0.00008
Age	− 0.09	0.05	− 2.01	0.044*	− 0.188, − 0.002	− 2.4e−06
Education (ref. no education)						
Primary school	0.93	1.19	0.79	0.431	− 1.39, 3.27	
Secondary school	3.53	1.14	3.11	0.002**	1.29, 5.760	
College and above	6.19	1.35	4.58	0.000***	3.54, 8.84	0.00006
Occupation(ref. unemployed)						
Self-employees	0.55	0.85	0.64	0.521	− 1.12, 2.21	
Civil servants	3.43	1.09	3.14	0.002**	1.28, 5.58	0.00005
Family size	0 .34	0.28	1.19	0.235	− 0.22,0.89	
Marital status (ref. other than married)						
Married	0.38	0.94	0.40	0.687	− 1.47, 2.23	
Solid waste generated (ref. < 1 sack)						
≥ 1 sacks	1.74	0.79	2.20	0.028*	0.19, 3.29	0.00004
Distance of dump site (ref. near)						
Far	1.58	0.57	2.75	0.006**	0.45, 2.72	0.00004
Awareness (ref. poor awareness)						
Good awareness	0.12	1.25	0.09	0.927	− 2.33, 2.56	
Homeownership (ref. No)						
Yes	0.59	0.80	0.73	0.463	− 0.99,2.17	
Time stay in the Town	0.005	0.04	0.14	0.891	− 0.067, 0.077	
Perceived satisfaction (ref. dissatisfied)						
Satisfied	3.89	0.58	6.67	0.000***	2.75, 5.05	0.0001
Wealth status (ref. poor)						
Medium	0.74	0.74	0.99	0.322	− 0.72, 2.20	
Rich	2.43	0.72	3.35	0.001**	1.009, 3.86	0.00003

* Significant with p-value ≤ 0.05

** Significant with p-value ≤ 0.01

*** Significant with p-value ≤ 0.001

### Discussion

The study showed that 81.06% of the participants were willing to pay for the service which was lower than studies done in other parts of Ethiopia and Nigeria about WTP for ISWM which were 92% and 87%, respectively [8, 13]. This might be due to differences in study setting. However, this study result was in line with a study done in Ethiopia (Jimma town) which was 83.5% [12], but higher than studies done in Tanzania 63% [14], South east Nigeria 64.4% [15], Ghana 57% [16], Nepal 61% [17] and India 63% [18]. The possible reason might be due to difference in study areas, period, design and demography.

The average amount of money respondents were WTP was 29.7 ETB ($1.07) which is higher by 10 ETB than the current fee. This shows households have an interest to contribute for environmental service, even more than the existing fee. The result of this study was higher than a study done in Nepal ($0.72), Ethiopia (Jimma and Mekelle) which were 17.26 Birr and 11.89 Birr [8, 12, 17], and lower than a study done in Ghana (Kumasi metropolis) which is $1.74 [16]. This might be due to the difference in study time and socio economic like per capita income of Ethiopia $2161, Nepal $2679 and that of Ghana is $4729 [19].

The study reveals that sex of household heads (being male) had statistically significant association with WTP. This finding is supported by a study conducted in China [20]. The possible explanation might be females had less economic decision power than males.

The household wealth status had statistically significant association with the WTP. This is similar to studies done in different parts of the world such as Ethiopia [8, 12], Nigeria [15], Nepal [17, 21] and Ghana [22].This might be due to the fact that a consumer with higher wealth status has a greater demand for waste management and more WTP.

This study showed that educational status of household heads had significant association with WTP. This is consistent with studies conducted in Ethiopia [8, 12] and Nigeria [23, 24]. The possible explanation may be due to the fact that educated people can understand easily the consequences of mismanagement of waste.

This study also showed that age of participants was negatively associated with WTP which was consistent with finding of studies done in Ethiopia [8], Nigeria [23], and Uganda [22]. The possible explanation may be those who are younger are more exposed to different health related information.

The amount of solid waste generated had significant relationship with the WTP for SWM. This finding was consistent with studies conducted in Tanzania and Ghana (Kumasi) [14, 16].This could be due to difficulties to dispose larger amount of waste and it needs high cost.

Distance from solid waste dumping sites had a positive relationship with the participants’ WTP for SWM service. This is similar with studies conducted in Ghana (Kumasi and Tema) [16, 25]. This is because in the longer the distance the more complicate problem of SWM as people would have to walk along distances to dispose waste.

This study also reveals that participants ‘perceived satisfaction with the service had a positive relationship with WTP for SWM. This result is similar with studies conducted in Ethiopia [12, 26]. This may be due to the rational behavior of customers, as their interest is maximizing utility or consumers are willing to utilize and pay for those services and goods that maximize their utility [27].

## Limitation of the study

Even though this study lies on the large sample size with high response rate (93%) due to our effort to include all households; the study has on limitation response biases which may overestimate or underestimate the results of WTP due to the use of self-reporting. The possibility of bias related to CVM and relied on cross-sectional data, restricting our ability to infer the causal directions underlying the observed associations.

## Additional file

**Additional file 1: Figure S1.** In this study, among household heads who participated in this study 81.06% (95% CI 78.5, 83.6) were willing to pay for improved solid waste management service, From which 22.4% of them were willing to pay 30 birr, The mean (± SD) amount of money household heads willing to pay was 29.7 (95% CI 29.08, 30.37) ETB (± 8.89) per month or 1.07 $USD. Accordingly, as the premium level decreases the probability to pay for the improved solid waste management service increase. At low premium levels nearly, all study participants were willing to pay that premium or vice versa (Additional file 1: Figure S1). The maximum amount of money household’s willingness to pay for improved solid waste management service in Injibara town, North West Ethiopia, 2018.

## Data Availability

Data will be available upon request from the corresponding author.

## Abbreviations

CVM: contingent valuation method
CI: confidence interval
ETB: Ethiopian Birr
ISWM: improved solid waste management
MWTP: maximum willingness to pay
SPSS: Statistical Package for Social Science
SWM: solid waste management
US$: United States Dollar
WTP: willingness to pay
