# Supplementary figures and images for: Willingness to pay for improved solid waste management and associated factors among households in Injibara town, Northwest Ethiopia
Source: BMC Res Notes. 2019 Jul 12;12:401. doi: 10.1186/s13104-019-4433-7 (PMC6626345; doi:10.1186/s13104-019-4433-7)

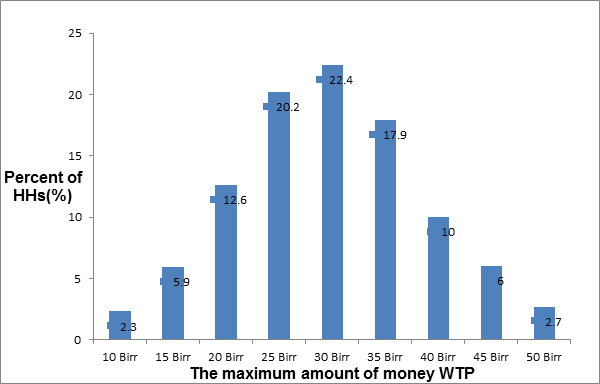

Supplement: Supplementary file 1 — Additional file 1: Figure S1. In this study, among household heads who participated in this study 81.06% (95% CI 78.5, 83.6) were willing to pay for improved solid waste management service, From which 22.4% of them were willing to pay 30 birr, The mean (± SD) amount of money household heads willing to pay was 29.7 (95% CI 29.08, 30.37) ETB (± 8.89) per month or 1.07 $USD. Accordingly, as the premium level decreases the probability to pay for the improved solid waste management service increase. At low premium levels nearly, all study participants were willing to pay that premium or vice versa (Additional file 1: Figure S1). The maximum amount of money household’s willingness to pay for improved solid waste management service in Injibara town, North West Ethiopia, 2018. [file 13104_2019_4433_MOESM1_ESM.tif]
